# The Book World of Medicine and Science

**Published:** 1893-07-08

**Authors:** 


### THE BOOK WORLD OF MEDICINE AND SCIENCE.

### BOOK REVIEWS.

THE TRAVELLERS A B C MEDICAL AND SURGICAL GUIDE. Second edition. (Published by Burroughs, Wellcome, and Co., London.)

A little knowledge is a dangerous thing, whether, therefore, the Travellers A B C Medical and Surgical Guide is to be seriously recommended to intending travellers by a journal which professes to have at heart the welfare of the public in matters medical and hygienic is a question not to be lightly dismissed. From a careful perusal and study of the subject matter of the 120 closely-written pages of this work, we conscientiously believe that in the hands of a person possessed of an average share of common sense, a patient suffering from a simple and straightforward medical ailment or surgical lesion might derive decided advantage to himself in cases where the assistance of a properly qualified man had to be dispensed with. When large blood vessels have been involved or long bones fractured, by following the directions given in the pages the most timid person might have the satisfaction of saving life, or preventing the life-long inconvenience of a badly-set, stiff, or shortened limb. It is certainly in matters surgical rather than medical that we should recommend reliance on this work, beyond the administration of a few safe drugs, such as quinine or iron, or an occasional purge, medical cases had better be left alone than treated on half knowledge. With these few reservations we are pleased to add the weight of our recommendation to a work which, though brought out partly by way of advertisement, has been issued by a firm which has done yeoman service in studying the comfort of the travelling public by reducing the weight of their medicinal impedimenta, and by invariably supplying drugs of first-rate quality. Their public services in these respects well deserve our recognition, which we take this opportunity of offering.

MENTAL DISEASES. By HENRY PUTNAM STEARRIS, A.M., M.D., Physician Superintendent of the Hartford Retreat, Lecturer on Mental Diseases in Yale University, &c. (Blakeston, Son, and Co.)

We can with confidence recommend this book to the student of medicine who is entering upon the study of mental diseases for the first time. The author states in his preface that it is for the use of such that these lectures were prepared. It is not a work for the specialist to consult. If he should refer to its pages for information it is probable that he will be disappointed in the results of his search. It is only what it pretends to be, "a basis for instruction in mental diseases." Without going into needless and confusing details of nomenclature, Dr. Stearris has given a good broad idea of the leading types of insanity, and he has the rare merit (among psychologists) of being able to express his meaning in language which is plain and easily understood. The usefulness of the book to the readers for whom it is intended would, in our opinion, have been increased had less space been devoted to the details of particular cases and more information given as to the methods of treatment to which allusion is made. As an example we may mention in the remarks upon the importance of regular physical exercise the reference to the use of music in arousing the attention and inducing

THE BOOK WORLD OF MEDICINE AND SCIENCE.—(*continued*).

muscular movements in a certain class of patients. When it is considered how varied are the symptoms in different cases of insanity of the same type and even in the same individual at different times it is doubtful whether any good purpose is served by giving so fully the accounts of single cases and their peculiarities. In an appendix are given extracts from the laws of the different States relating to the care of the insane. They are interesting, but we do not find much that we wish to appropriate and add to our own Lunacy Act. This does not by any means imply that we look upon that effort of the Legislature as perfect. The following extract from the laws of South Carolina, 1884, is interesting: "Physicians giving certificates recommending commitment to the asylum of a person who is simply idiotic, epileptic, physically infirm, or mentally imbecile, unless such person is violent or dangerous, shall be deemed guilty of a misdemeanour, and upon conviction thereof shall be fined by the District Court." What would be the effect of such a clause in our Act? Would it diminish the number of hopeless, helpless, bedridden dements sent to institutions for the insane, who might as well be nursed in the wards of the workhouse infirmary as in the already often crowded sick room of the asylum? We fear not. It is more probable that the immediate result would be an apparent outburst of violent and dangerous propensities among these wrecks of humanity. The recovery rate in the asylums of the past ought to have been high. Though the treatment in them was far behind what it is in similar institutions of to-day, they must have had a greater proportion of curable cases amongst their admissions. The wards of a public asylum of to-day are made to an extent that was never intended—mere lumber rooms for human wreckage.

A Contribution to the Pathology of the Vermiform Appendix. By T. N. Kelynack, M.D., Pathologist to the Manchester Royal Infirmary; Demonstrator and Assistant Lecturer on Pathology in the Owens College. With Illustrations and Bibliography. (London: H. K. Lewis. 1893.)

This essay was prepared as a dissertation, to be presented to the Victoria University for the degree of M.D., and it must in all fairness be judged from that standpoint. We may at once say that it is fully up to the standard of excellence required at any university. It is the outcome of very careful and diligent study of the writings of others, and when we mention that the bibliography at the end occupies more than 60 pages, it is at once evident how full Dr. Kelynack's search into the literature of his subject has been. Not only does the reader find much of great interest that Dr. Kelynack has gathered himself from this source, but all workers in this field will find such a very full bibliography of the greatest service to them in their work, and if this volume contained nothing more, its author would have rendered his profession no mean service. But there is more. Until quite recently the vermiform appendix was treated with studied neglect, no attention was paid to it, and as a consequence little or nothing was known about it. Then quite suddenly it was recognised that this so-called useless vestige was, at any rate, of great clinical and pathological importance, as being the seat of grave inflammatory and other troubles. Surgeons paid great attention to it, and it was the subject of very frequent operations. This

THE BOOK WORLD OF MEDICINE AND SCIENCE—(*continued*).

"discovery" of the vermiform appendix has necessitated a careful study of the part itself. For many of those who have written upon it from the clinical and pathological standpoint, have been led into error by being unaware of the normal anatomy of the parts concerned, Dr. Kelynack has availed himself of his opportunities as pathologist of the Royal Infirmary, Manchester, to study specially the normal anatomy of the cœcum and appendix, the lower end of ileum, and the surrounding peritoneum. The results of this study are before us, and his description of the peritoneal folds and fossæ about the appendix, and of its various positions, are very valuable. In much he has to corroborate the statements of others, but in much he has to correct them, and all who desire to know the normal anatomy of this part and its more common variations, will find great assistance from this work. The clinical part of the essay is, perhaps, less interesting and valuable, for here the author speaks chiefly of the experience of others, while in the anatomical part he tells us his own.

